# Dexmedetomidine improves attention and recall in agitated critically ill patients

**DOI:** 10.1186/cc9775

**Published:** 2011-03-11

**Authors:** MM Mirski, RG Gill, PM Murakami, CT Thompson, JL Lewin

**Affiliations:** 1 Johns Hopkins Medicine, Baltimore, MD, USA

## Introduction

It is of clinical interest to maintain patient comfort in the ICU and yet preserve their intellectual function, arousal and interaction. Recently, dexmedetomidine (DEX) was demonstrated in the ANIST Trial to preserve intellectual function as compared with propofol (PRO) when used as conscious sedation in both agitated neurologically intact and brain-injured critically ill patients [1]. The purpose of this study was to further understand whether selective areas of cognition were specifically affected by PRO and DEX through sub-analysis of the Trial's results on each of the five subscales of the Adapted Cognitive Exam (ACE).

## Methods

We preformed a *post-hoc *analysis of the prospective randomized, double-blinded cross-over designed ANIST trial that compared cognitive differences between PRO and DEX on the validated 100-point Hopkins ACE. This current study further investigated differences by analyzing the five subscales of the ACE, which consist of Orientation, Language, Registration, Attention/Calculation and Recall. Analysis included a generalized estimating equations approach to estimate differences between drugs while accounting for within-subject correlation arising from the crossover design. We examined unadjusted and adjusted models both with and without inclusion of potential period effects. We also accounted for period effects, and robust variance estimates were used to calculate standard errors.

## Results

Sedation with PRO diminished adjusted scores on four of the ACE subscales (*P *< 0.01), while DEX improved adjusted scores on two of the subscales (Attention/Calculation 2.35, 95% CI: 0.11 to 4.59; Recall: 2.03, 95% CI: 0.03 to 4.04). The other estimates for the effects of PRO and DEX on the ACE subscales were not statistically significant using a significance level of 0.05. The positive and significant difference in the change of ACE score between DEX versus PRO held up in all of the subscales.

## Conclusions

Our findings indicate that DEX not only preserved but also improved Attention/Calculation and Recall in ICU patients who were awake, agitated and required sedation. This was evident by higher mean ACE subscale scores when compared with their baseline. Our findings suggest that DEX improved overall cognitive function without significantly compromising the ability to focus and recall events.
